# Reviewer acknowledgement 2013

**DOI:** 10.1186/1471-2466-14-13

**Published:** 2014-02-14

**Authors:** Catia Cornacchia

**Affiliations:** 1 BioMed Central, Floor 6, 236 Gray's Inn Road, London, WC1X 8HB, United Kingdom

## Abstract

**Contributing reviewers:**

The editors of BMC Pulmonary Medicine would like to thank all our reviewers who have contributed to the journal in Volume 13 (2013).

## 

Adel Mansur

UK

Adrian Loerbroks

Germany

Afroditi Boutou

Greece

Ahmed Fahim

UK

Ajanta Sharma

India

Alastair Innes

UK

Alberto Giannoni

Italy

Alessandro Marcon

Italy

Alex Mackay

UK

Alex Sánchez-Pla

Spain

Alf Tunsäter

Sweden

Amir Hossein Jafarian

Iran

Ana De Paul

Argentina

Andras Rosztoczy

Hungary

Andrea Melani

Italy

Andreas Rembert Koczulla

Germany

Andrew Wilson

UK

Angela Koutsokera

Switzerland

Annemarie Lee

Australia

Antonia Koutsoukou

Greece

Antonio Pereira-Vega

Spain

Antonio Corrado

Italy

Argyris Tzouvelekis

Greece

Aryeh Fischer

USA

Arzu C Yorgancioglu

Turkey

Asiye Kanbay

Turkey

Aurélie Cazes

France

Bandana Saini

Australia

Benjamin Fox

Israel

Benoit Arnould

France

Bertrand Renaud

France

Borja G Cosio

Spain

Bouchra Lamia

France

Brenda Morrow

South Africa

Brian Wickes

USA

Brigitte Fauroux

France

Bruno Crestani

France

Carl Lombard

South Africa

Carolyn D'Ambrosio

USA

Carrie Breton

USA

Catherine Byrnes

New Zealand

Catherine Greene

Ireland

Celeste Porsbjerg

Denmark

Celso R F Carvalho

Brazil

Chantal Raherison

France

Charles Burger

USA

Chin-Chung Shu

Taiwan

Chris Smerecnik

Netherlands

Christian Becker

USA

Christian Clarenbach

Switzerland

Christian Wejse

Denmark

Christina Alexopoulou

Greece

Christoph Schranz

Switzerland

Christophe Marguet

France

Dai Lu-Ming

China

Daisy Janssen

Netherlands

Dajun Deng

China

Daniel Culver

USA

Dave Singh

UK

David Jimenez

Spain

David Mannino

USA

Denis O'Donnell

Canada

Dermot Ryan

UK

Desmond Murphy

Ireland

Diane Carroll

USA

Diederik Gommers

Netherlands

Dieudonnée Togbe

France

Dmitri Pechkovsky

Canada

Don Poldermans

Netherlands

Donal O'Donoghue

UK

Donald Tashkin

USA

Donna Rennie

Canada

Eddy Fan

Canada

Edoardo Savarino

Italy

Edward Kerwin

USA

Edwin Roger Parra

Brazil

Elif Cadirci

Turkey

Elisabeth Svensson

Sweden

Elizabeth Wasilevich

USA

Elliott Dasenbrook

USA

Eric Snyder

USA

Erica Rutten

Netherlands

Esmaeil Mortaz

Netherlands

Esther Julian

Spain

Esther Barreiro

Spain

Evan Brittain

USA

Ewa Wysocka

Poland

Fabiano Di Marco

Italy

Fa-Yauh Lee

Taiwan

Fekri Abroug

Tunisia

Frances De Man

Netherlands

Francesco Pistelli

Italy

Francis Gilchrist

UK

Francisco García-Río

Spain

Francisco-Javier Gonzalez-Barcala

Spain

Francois Stephan

France

Frede Donskov

Denmark

Gabriela Godaly

Sweden

Gaetan Deslee

France

Geertjan Wesseling

Netherlands

Geoff Chase

New Zealand

George Konstantinou

USA

Ghislaine Gayan-Ramirez

Belgium

Gilles Hejblum

France

Giorgio Vescoo

Italy

Girdhar Agarwal

India

Gomez Roman Jose Javier

Spain

Guelmisal Gueder

Germany

Haralampos Moutsopoulos

Greece

Hartmut Grasemann

Canada

Hendrik Jan Ankersmit

Austria

Hermann Wrigge

Germany

Hiroshi Iwasaki

Japan

Hisako Matsumoto

Japan

Hitesh Batra

USA

Hossein Kakooei

Iran

Ian Yang

Australia

Ignacio Gil-Bazo

Spain

Ingrid Du Rand

UK

In-Young Yoon

South Korea

Irma Godoy

Brazil

Jaishree Bhosle

UK

James Donohue

USA

Jan Hendrik Storre

Germany

Jan Peder Amlie

Norway

Janwillem Kocks

Netherlands

Jaya Prakash Sugunaraj

USA

Jean-Paul Janssens

Switzerland

Jeffrey Swigris

USA

Jens Geiseler

Germany

Jens Bräunlich

Germany

Jeremy Road

Canada

Jianzhu Chen

USA

Jiri Widimsky

Czech Republic

Joana Mascarenhas

Portugal

Joanna Stewart

New Zealand

Joanne Murphy-Ullrich

USA

Joao Durigan

Brazil

Johanna Williams

UK

John Ludbrook

Australia

John Kalbfleisch

USA

Jonathan Hobson

UK

José Luis Izquierdo Alonso

Spain

Josh Torgovnick

USA

Josuel Ora

Italy

Judith Garcia-Aymerich

Spain

Julia Walters

Australia

Justin Travers

New Zealand

Kaid Darwiche

Germany

Kamel Hamzaoui

Tunisia

Karina Portillo Carroz

Spain

Karl-Josef Franke

Germany

Kenneth Casey

USA

Kerstin Ström

Sweden

Kevin Webb

UK

Kiran Shekar

Australia

Konstantinos Kostikas

Greece

Kosaku Komiya

Japan

Kyndaron Reinier

USA

Laima Taraseviciene-Stewart

USA

Laurie Whittaker

USA

Lei Wan

Taiwan

Leila D Amorim

Brazil

Leonardo Trasande

USA

Lewis Rubin

USA

Li Zuo

USA

Lisa Wood

Australia

Lisa Edwards

USA

Lisa Schwiebert

USA

Lucie Blais

Canada

Luis Nannini

Argentina

Luis Valdés

Spain

Luis Felipe Flores-Suarez

Mexico

Maarten Van Den Berge

Netherlands

Maher Khdour

Palestinian Territory

Marcello Verini

Italy

Marco Contoli

Italy

Margo Benoit

USA

Mark Dransfield

USA

Mark Weatherall

New Zealand

Markus A Rose

Germany

Martin Liu

USA

Maryline Bonnet

Switzerland

Masanori Yasuo

Japan

Masaya Takemura

Japan

Masayuki Hanaoka

Japan

Massimo Antonelli

Italy

Massimo Miniati

Italy

Massimo Corradi

Italy

Massimo Pistolesi

Italy

Matthew Foster

USA

Matthew Rank

USA

Maurizio Luisetti

Italy

May Brit Lund

Norway

Melissa Piper

USA

Melissa Roberts

USA

Michał Ciurzyński

Poland

Michael Soussan

France

Michael Morgan

UK

Michael Seear

Canada

Michael Westhoff

Germany

Michael Stickland

Canada

Michael Lu

USA

Mieke Dentener

Netherlands

Mike Sathekge

South Africa

Milica Kontic

Serbia

Mitja Lainscak

Slovenia

Muhammad Towhid Salam

USA

Murugesan Rajaram

USA

Neil Alexis

USA

Niaz Banaei

USA

Nicholas Glanville

UK

Nicholas Kenyon

USA

Nicolas De Prost

France

Niki Reynaert

Netherlands

Nils Nickel

Germany

Nir Osherov

Israel

Norman Morris

Australia

Olaf Holz

Germany

Olaf Eickmeier

Germany

Olufemi Desalu

Nigeria

P Marco Fisichella

USA

Paola Michelozzi

Italy

Paolo Montuschi

Italy

Patricia Silveyra

USA

Patrick Murphy

UK

Patrudu Makena

USA

Peter Kardos

Germany

Peter Spieth

Germany

Phil Hansbro

Australia

Pierachille Santus

Italy

Pierre-Olivier Bridevaux

Switzerland

Pierre-Regis Burgel

France

Pinar Karaca-Mandic

USA

Rainer Gloeckl

Germany

Ralph Panos

USA

Raul Dela Cadena

USA

Raymond Ten Eyck

USA

Renda Wiener

USA

Robert Freishtat

USA

Robert P Young

New Zealand

Roberto G Carbone Md, Fccp

Italy

Robin Condliffe

UK

Rodolfo Paula Vieira

Brazil

Rosemary Barnes

UK

Rui Baptista

Portugal

Sally Chappell

UK

Sanjay Chotirmall

Ireland

Sara Maio

Italy

Scott Sands

USA

Scott Evans

USA

Sejal Saglani

UK

Shan Zienolddiny

Norway

Sharon Rosenberg

USA

Shigeo Muro

Japan

Shin Matsuoka

Japan

Shinya Makino

Japan

Shyam-Lung Lin

Taiwan

Sidney Braman

USA

Silvia Bielsa

Spain

Silvia Ulrich

Switzerland

Soo-Keol Lee

South Korea

Spyros Mentzelopoulos

Greece

Spyros Papiris

Greece

Stephan Walterspacher

Germany

Stephanie Shore

USA

Stephanie Taylor

UK

Stephen Frohlich

Ireland

Stephen Bourke

UK

Steven Kawut

USA

Steven Hollenberg

USA

Stylianos Orfanos

Greece

Susan Janson

USA

Susanne Schulz

Germany

Tadashi Ishida

Japan

Takashi Nojiri

Japan

Takeharu Koga

Japan

Tamera Corte

Australia

T-C Lee

Taiwan

Terence Seemungal

Trinidad and Tobago

Thomas Janssens

Belgium

Thomas Staudinger

Austria

Tibor Wittmann

Hungary

Tim Mcmahon

USA

Tobias Breidthardt

Switzerland

Toby Maher

UK

Tomasz Golczewski

Poland

Toru Oga

Japan

Ulas Bagci

USA

Valerie Martinez

France

Violeta Vucinic

Serbia

Virginia Carrieri-Kohlman

USA

Vlasis Polychronopoulos

Greece

William Gower

USA

Yan Tian

USA

Yoshiki Demura

Japan

Yoshinosuke Fukuchi

Japan

Yuben Moodley

Australia

Yuji Tohda

Japan

## Pre-publication history

The pre-publication history for this paper can be accessed here:

http://www.biomedcentral.com/1471-2466/14/13/prepub

